# Combination of Melatonin and Zoledronic Acid Suppressed the Giant Cell Tumor of Bone *in vitro* and *in vivo*

**DOI:** 10.3389/fcell.2021.690502

**Published:** 2021-08-10

**Authors:** Xudong Wang, Peiqiang Su, Yan Kang, Caixia Xu, Jincheng Qiu, Jinna Wu, Puyi Sheng, Dongsheng Huang, Ziji Zhang

**Affiliations:** ^1^Department of Orthopedics, The First Affiliated Hospital of Sun Yat-sen University, Guangzhou, China; ^2^Guangdong Provincial Key Laboratory of Orthopedics and Traumatology, The First Affiliated Hospital of Sun Yat-sen University, Guangzhou, China; ^3^Research Centre for Translational Medicine, The First Affiliated Hospital of Sun Yat-sen University, Guangzhou, China; ^4^Department of Orthopedics, Sun Yat-sen Memorial Hospital of Sun Yat-sen University, Guangzhou, China

**Keywords:** melatonin, zoledronic acid, giant cell tumor of bone, proliferation, apoptosis, migration, invasion

## Abstract

Melatonin (Mlt) confers potential antitumor effects in various types of cancer. However, to the best of our knowledge, the role of Mlt in the giant cell tumor of bone (GCTB) remains unknown. Moreover, further research is required to assess whether Mlt can enhance the therapeutic effect of zoledronic acid (Zol), a commonly used anti-GCTB drug. In this research, we investigated the effects of Mlt, Zol, and the combination of these two drugs on GCTB cells’ characteristics, including cell proliferation, apoptosis, osteogenic differentiation, migration, and invasion. The cell counting kit-8 (CCK-8) assay, colony formation assay, terminal deoxynucleotidyl transferase-mediated dUTP nick-end labeling assay (TUNEL), alkaline phosphatase (ALP) staining, alizarin red staining (ARS), scratch wound healing assay, and transwell experiment were performed, respectively. Our results showed that Mlt could effectively inhibit the proliferation, migration, and invasion of GCTB cells, as well as promote the apoptosis and osteogenic differentiation of tumor cells. Of note, a stronger antitumor effect was observed when Mlt was combined with Zol treatment. This therapeutic effect might be achieved by inhibiting the activation of both the Hippo and NF-κB pathways. In conclusion, our study suggests that Mlt can be a new treatment for GCTB, which could further enhance the antitumor effect of Zol.

## Introduction

Giant cell tumor of bone (GCTB) is a benign but aggressive bone tumor, which has the potential to destroy the bone and invade into the surrounding soft tissue. This leads to local pain and metastasis (Szendroi, 2004; Alberghini et al., 2010; Verschoor et al., 2018). At present, surgical resection is the primary treatment for GCTB; however, this treatment is not applicable in some sections of the body, such as the skull, pelvis, and spine. Unfortunately, 15–50% patients still experience local tumor recurrence after surgical treatment (Szendroi, 2004; Xu S. F. et al., 2013; van der Heijden et al., 2014). For patients with unresectable, residual, or recurring GCTB, some adjuvant therapies, such as radiotherapy, chemotherapy, drugs (including zoledronic acid and denosumab), and embolization therapy, may be prescribed. However, these methods are less effective, accompanied by high cost and side effects (Gaston et al., 2011). Therefore, new treatments for GCTB are urgently needed.

Melatonin (Mlt), a neuroendocrine hormone primarily secreted by the pineal gland, exhibits a wide range of physiological functions, such as improving sleep and promoting poly-differentiation of stem cells, and is an antiaging, antioxidative, and antiosteoporotic agent (Reiter et al., 2016; Nopparat et al., 2017; Gao et al., 2018; Sharma et al., 2018; Qiu et al., 2019; Wang et al., 2019; Wang et al., 2021). In recent years, the therapeutic effect of Mlt on tumors, including lung cancer, liver cancer, and breast cancer, has received great attention (Ordonez et al., 2015; Borin et al., 2016; Ma et al., 2019) however, the effect of Mlt on GCTB remains unknown.

Because Mlt has displayed fewer side effects with a relatively lower cost than that of other tumor treatments, we explored the therapeutic effect of Mlt on GCTB through *in vitro* and *in vivo* experiments in this study. Moreover, we explored whether Mlt can enhance the therapeutic effect of the commonly used anti-GCTB drug zoledronic acid (Zol).

## Materials and Methods

### Cell Culture

Human GCTB cells were purchased from Beijing Beina Chuanglian Biotechnology Institute (Beijing, CN), and the cells were cultured in Roswell Park Memorial Institute (RPMI) 1640 medium containing 10% fetal bovine serum (FBS), 100 mg/mL streptomycin, and 100 U/mL penicillin. The culture medium was changed every 2 days. The cells were cultured in an incubator at 37°C with 5% CO_2_.

### Antibodies and Reagents

Antibodies against osteopontin (OPN), osteocalcin (OCN), P-LATS 1/2, LATS 1, LATS 2, P-yes-associated protein (YAP), YAP, P-P65,P65, Lamin B1, P-IκBα, IκBα, CYCLIN B1, PCNA, KI-67, CLEAVED CASPASE-3, CASPASE-3, BAX, and BCL2 were purchased from Abcam (Cambridge, United Kingdom). Antibody against glyceraldehyde-3-phosphate dehydrogenase (GAPDH) and runt-related transcription factor-2 (RUNX2) was purchased from CST (Boston, United States). Mlt and Zol were purchased from MCE (New Jersey, United States).

### Cell Counting Kit-8 Assay

After treatment, the cell proliferation was detected using the cell counting kit-8 (CCK-8) assay (#ck3404, LABFOM, Guangzhou, China) as instructed by the manufacturer. In brief, GCTB cells were digested by trypsin–ethylenediamine tetraacetic acid (EDTA) (0.25%) (#25200056, GIBCO, Guangzhou, China) and then seeded into a 96-well plate at a density of 4 × 10^3^ cells per well. After 6, 12, 24, 48, and 72 h, CCK-8 was added to detect the proliferation of GCTB cells *via* Elx800 (BioTek, Winooski, Vermont, United States) at 450 nm spectrophotometry.

### Colony Formation Assay

After Mlt or/and Zol treatment, cell proliferation was detected *via* a colony formation assay. In brief, GCTB cells were digested by trypsin-EDTA (0.25%) and then seeded into a 6-well plate at a density of 1 × 10^3^ cells per well. After 14 days, 0.1% crystal violet solution was added to detect the proliferation of GCTB cells. The culture plate was then scanned with a gross scanning instrument (Bio-rad, GS-800, Hercules, CA, United States).

### Terminal Deoxynucleotidyl Transferase-Mediated dUTP Nick-End Labeling Assay

After treatment, the apoptosis rate of GCTB cells was detected with the terminal deoxynucleotidyl transferase-mediated dUTP nick-end labeling (TUNEL) assay (#GB17002, GUGE, Wuhan, China), as per manufacturer’s instructions. In brief, the cells were digested by trypsin-EDTA (0.25%) and then seeded into a 6-well plate at a density of 4 × 10^4^ cells per well. After treatment, the TUNEL assay was used to detect the apoptosis of GCTB cells. TUNEL-positive cells were photographed using a microscope. At least four images of 200 × magnification were captured at random for each group. The number of TUNEL-positive cells in each image was quantified using Image-Pro Plus 6.0, and the percentage of TUNEL-positive cells relative to propidium iodide (PI)-stained cells was calculated.

### Alkaline Phosphatase Staining and Alizarin Red Staining

The expression of alkaline phosphatase (ALP) in GCTB cells was detected by using the BCIP/NBT ALP color development kit (#B3679; Sigma), as per manufacturer’s instructions. To evaluate osteogenic differentiation, the cells were also stained with an alizarin red reagent (#G1450; LIUHEBIO) for 5 min at room temperature. Finally, ALP and alizarin red staining (ARS) in the cells were observed by gross scanning (Bio-rad, GS-800, Hercules, United States).

### Scratch Wound Healing Assay

After the cells were cultured on 6-well plates to a monolayer, a wound was created with a 20-μL pipette tip, ensuring that all scratch widths were consistent. Then, the cells were washed with phosphate-buffered saline (PBS) three times, and the medium was replaced with different treatments (GCTB cells were treated with drugs (Mlt or/and Zol) during wound healing assay but not pretreated cells with drugs). Then, the cells were cultured in an incubator at 37°C with 5% CO_2_. Cells were photographed after 0, 12, 24, 48, and 72 h, and the scratch width was measured using the Photoshop software.

### Transwell Invasion and Migration Assay

Matrigel was diluted using FBS-free RPMI 1640 medium in a 1:8 ratio and coated on the upper chamber of the bottom membrane of transwell chambers, followed by incubation at 37°C for 30 min. After 24 h cell starvation, the cells were digested, and the cell density was adjusted to 2 × 10^5^/mL. Then, 100 μL cell suspension was added to the transwell chamber, and 600 μL medium containing 20% FBS was added to the lower chamber. After the cells were cultured for 24 and 48 h and after removing non-migrated cells and Matrigel with wet cotton balls from the upper compartment, 0.1% crystal violet staining of the cells was performed for 30 min as per the standard staining procedure and counted. The cells’ transwell migration assay was performed in the same manner as that for the invasion assay but without the procedure of Matrigel coating.

### Quantitative Reverse Transcription Polymerase Chain Reaction and Real Time PCR

Total RNA samples from cultured GCTB cells or mouse tumors were prepared using the total RNA extraction kit (#15596018; Invitrogen, Guangzhou, China), as per manufacturer’s instructions. RNA concentrations were measured using NanoDrop 2000 instrument (Thermo Fisher Scientific, United States). The cDNA for quantitative reverse transcription polymerase chain reaction (qRT-PCR) was subsequently synthesized from 1 μg total RNA using Evo M-MLVRT kit (#AG11706; Accurate Biotechnology, Hunan, China), according to the manufacturer’s instructions. Then, the qRT-PCR assay was performed using the LightCycler 480 SYBR Green I Master Kit (#4887352001-1; Roche, Guangzhou, China), as per the manufacturer’s instructions. The final expression levels of mRNAs were calculated by the standard 2^–ΔΔ*Ct*^ method based on at least three biological replicates. The primer sequences used for quantitation are listed in Table 1.

**TABLE 1:** The primer sequences used for quantitative PCR.

Gene	Primer sequence (5′–3′)
*GAPDH*	Forward: AGAAAAACCTGCCAAATATGATGAC;
	Reverse: TGGGTGTCGCTGTTGAAGTC;
*CYCLIN B1*	Forward: AATAAGGCGAAGATCAACATGGC;
	Reverse: TTTGTTACCAATGTCCCCAAGAG;
*PCNA*	Forward: CCTGCTGGGATATTAGCTCCA;
	Reverse: CAGCGGTAGGTGTCGAAGC;
*KI-67*	Forward: ACGCCTGGTTACTATCAAAAGG;
	Reverse: CAGACCCATTTACTTGTGTTGGA;
*BAX*	Forward: CCCGAGAGGTCTTTTTCCGAG;
	Reverse: CCAGCCCATGATGGTTCTGAT;
*BCL2*	Forward: GGTGGGGTCATGTGTGTGG;
	Reverse: CGGTTCAGGTACTCAGTCATCC;
*RUNX2*	Forward: AGAAGGCACAGACAGAAGCTTGA;
	Reverse: AGGAATGCGCCCTAAATCACT;
*OPN*	Forward: GCGAGGAGTTGAATGGTG;
	Reverse: CTTGTGCTGTGGGTTTC;
*OCN*	Forward: CACTCCTCGCCCTATTGGC;
	Reverse: CCCTCCTGCTTGGACACAAAG;
*E-cadherin*	Forward: CGAGAGCTACACGTTCACGG;
	Reverse: GGGTGTCGAGGGAAAAATAGG;
*N-cadherin*	Forward: TCAGGCGTCTGTAGAGGCTT;
	Reverse: ATGCACATCCTTCGATAAGACTG;
*Vimentin*	Forward: AGTCCACTGAGTACCGGAGAC;
	Reverse: CATTTCACGCATCTGGCGTTC;
*Snail*	Forward: TCGGAAGCCTAACTACAGCGA;
	Reverse: AGATGAGCATTGGCAGCGAG;
*Slug*	Forward: CGAACTGGACACACATACAGTG;
	Reverse: CTGAGGATCTCTGGTTGTGGT;
*MMP9*	Forward: TGTACCGCTATGGTTACACTCG;
	Reverse: GGCAGGGACAGTTGCTTCT;
*MMP13*	Forward: ACTGAGAGGCTCCGAGAAATG;
	Reverse: GAACCCCGCATCTTGGCTT;
*CCL2*	Forward: CAGCCAGATGCAATCAATGCC;
	Reverse: TGGAATCCTGAACCCACTTCT;

### Western Blot Analysis

Total protein samples were extracted from cultured GCTB cells using radioimmunoprecipitation (RIPA) assay buffer containing phosphatase and proteinase inhibitors (1:100). Cytoplasmic and nuclear proteins were extracted using a Minute^TM^ cytoplasmic and nuclear fractionation kit (Invent, United States). Protein concentrations were determined by a bicinchoninic acid protein assay kit (Beyotime, United States), and then the proteins were separated by 10% sodium dodecyl sulfate–polyacrylamide gel and blotted onto a polyvinylidene fluoride (PVDF) membrane. The membranes were blocked using 5% lipid-free milk solution for 1 h; incubated with anti-phospho-LATS 1/2, anti-LATS 1, anti-LATS 2, anti-phospho-YAP, anti-YAP, anti-p65, anti-phospho-P65, anti-IκBα, anti-phospho-IκBα, and anti-GAPDH antibodies overnight at 4°C; and then incubated with diluted secondary antibodies. Finally, the PVDF membranes were visualized with an enhanced chemiluminescent western blot detection system.

### Immunohistochemistry Analysis

Paraffin sections were prepared, and immunohistochemistry (IHC) was performed using Histostain-Plus kit. The primary antibodies included anti-CYCLIN B1, PCNA, KI-67, CLEAVED CASPASE-3, CASPASE-3, BAX, and BCL2 antibodies. A DAB horseradish peroxidase color development kit was used for detection, and the staining intensity was scored according to the previous article (Lian et al., 2019).

### Hematoxylin and Eosin Staining

Liver, spleen, kidney, and heart samples were fixed with 4% paraformaldehyde, dehydrated, embedded in paraffin, and cut into 5 μm slices, after which they were dewaxed by xylene and alcohol. After slices were immersed in distilled water, hematoxylin and eosin (HE) staining was performed according to the standard protocols.

### Animal Experiment

A total of 32 specific-pathogen-free (SPF) nude mice aged 6–8 weeks (16 female nude mice and 16 male nude mice) were purchased from Charles River Laboratories (Beijing, China). They were randomly classified into four groups: the Con group, Mlt group, Zol group, and Mlt and Zol combination group. Each group included four female nude mice and four male nude mice. The nude mice were housed in a 12-h light/12-h dark condition. The right groin area was selected as the subcutaneous tumor formation site of nude mice, where each nude mouse was injected with 100 μL tumor cell suspension (1 × 10^5^ tumor cells). After the tumors grew for 1 week, the nude mice were given an intraperitoneal injection of different drugs (50 mg/kg/d Mlt or 5 mg/kg/d Zol) for 5 weeks. Then, the long and short diameters of the tumors in each group of animals were measured once a week, and the tumor volume was calculated. After 5 weeks of drug administration, the tumor tissues of the mice were removed and weighed, followed by qRT-PCR and IHC experiments. This study was approved by the Forevergen Biosciences Experimental Animal Ethics Committee (Animal approval certificate information ID: IACUC-G16030).

### Statistical Analysis

All quantitative data have been presented as the mean ± standard and analyzed using SPSS 20.0 software. The differences between 2 and >2 groups were determined by the Student’s *t*-test and analysis of variance methods, respectively. *P* < 0.05 is statistically significant. Important notations are as follows: “^∗^” represents *P* < 0.05 vs. Con group, “^∗∗^” represents *P* < 0.01 vs. Con group, “^∗∗∗^” represents *P* < 0.001 vs. Con group, and “#” represents *P* < 0.05 vs. Zol group.

## Results

### Mlt, Zol, and the Combination of These Two Drugs Inhibit the Proliferation of GCTB Cells

**FIGURE 1:**
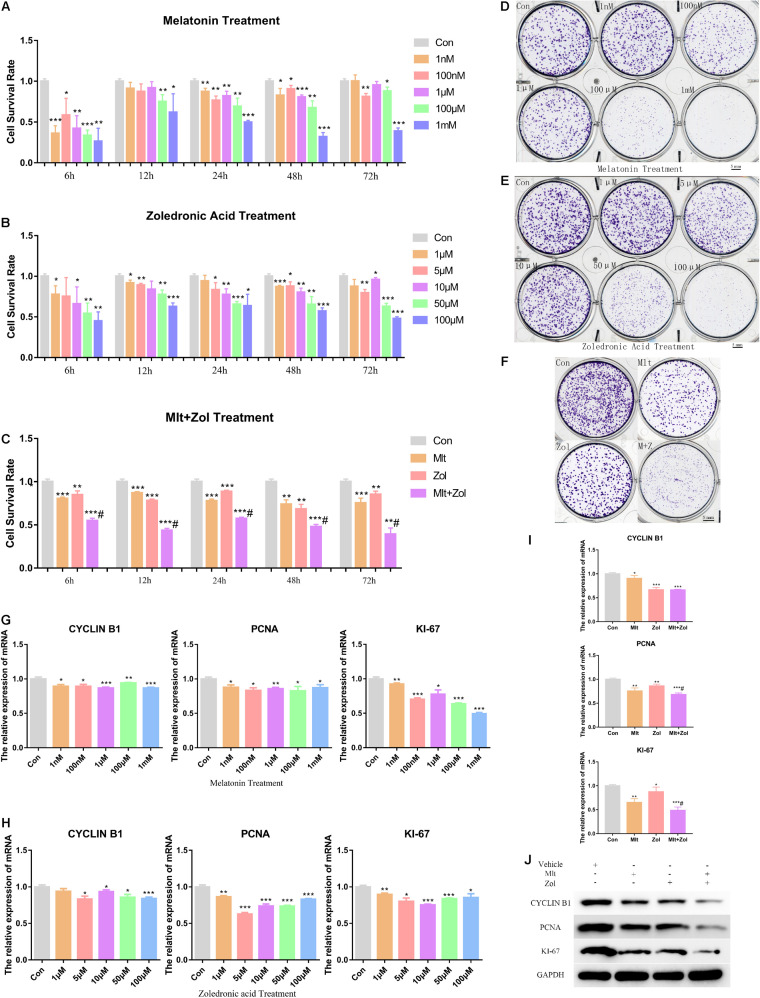
Mlt, Zol, and their combination inhibit the proliferation of GCTB cells. **(A)** The proliferation ability of tumor cells treated with Mlt at different concentrations (1 nM, 100 nM, 1 μM, 100 μM, and 1 mM) for 6, 12, 24, 48, and 72 h were detected *via* CCK-8 assay. **(B)** The CCK-8 assay was used to detect the proliferation of tumor cells treated with Zol at different concentrations (1, 5, 10, 50, 100 μM) for 6, 12, 24, 48, and 72 h. **(C)** The CCK-8 assay was used to detect the proliferation ability of tumor cells after treatment with Mlt, Zol, and Mlt + Zol at 6, 12, 24, 48, and 72 h. **(D)** The colony formation assay was used to detect the proliferation of tumor cells treated with Mlt at different concentrations (1 nM, 100 nM, 1 μM, 100 μM, and 1 mM) for 14 days. **(E)** The colony formation assay was used to detect the proliferation of tumor cells treated with Zol at different concentrations (1, 5, 10, 50, and 100 μM) for 14 days. **(F)** The colony formation assay was used to detect the proliferation of tumor cells after 14 days of treatment with Mlt, Zol, and Mlt + Zol. **(G)** Relative mRNA levels of proliferation markers’ genes (*CYCLIN B1*, *PCNA*, and *KI-67*) in human GCTB cells treated with Mlt were measured by qRT-PCR. **(H)** Relative mRNA levels of proliferation markers’ genes in human GCTB cells treated with Zol were measured by qRT-PCR. **(I,J)** Relative mRNA and protein levels of proliferation markers in human GCTB cells treated with Mlt, Zol, and Mlt + Zol were measured by qRT-PCR and western blot. Con: control; Mlt: melatonin; Zol: zoledronic acid; *P < 0.05 vs. Con, **P < 0.01 vs. Con, ***P < 0.001 vs. Con, #*P* < 0.05 vs. Zol. Scale bars: 5 mm.

We explored the effects of different Mlt concentrations (1 nM, 100 nM, 1 μM, 100 μM, 1 mM) and Zol concentrations (1, 5, 10, 50, 100 μM) on the proliferation of tumor cells. First, we used the CCK-8 assay for detecting tumor cell proliferation at 6, 12, 24, 48, and 72 h after treatment. We found that different concentrations of Mlt or Zol inhibited the proliferation of GCTB cells, and the effects of high concentrations were more obvious (Figures 1A,B). Moreover, the combination of the two drugs (1 mM Mlt and 100 μM Zol) showed stronger inhibitory effects (Figure 1C), and the combination index of Mlt and Zol showed that the combination of Mlt and Zol had a moderate synergistic effect (Supplementary Figure 1). We then performed the tumor colony formation assay and observed similar effects (the concentrations of Mlt and Zol were consistent with the drug concentrations used in CCK-8 assay) (Figures 1D–F). Finally, *via* the mRNA detection of proliferation markers *CYCLIN B1*, *PCNA*, and *KI-67*, we found that treatment with different concentrations of Mlt and Zol suppressed the mRNA expression of proliferation markers (Figures 1G,H), and the mRNA and protein detection of proliferation markers showed that the combination of the two drugs showed a stronger inhibitory effect (Figures 1I,J). In summary, Mlt inhibited the proliferation of GCTB and can further enhance the inhibitory effect of Zol.

### Mlt, Zol, and Their Combination Promote the Apoptosis of GCTB Cells

**FIGURE 2:**
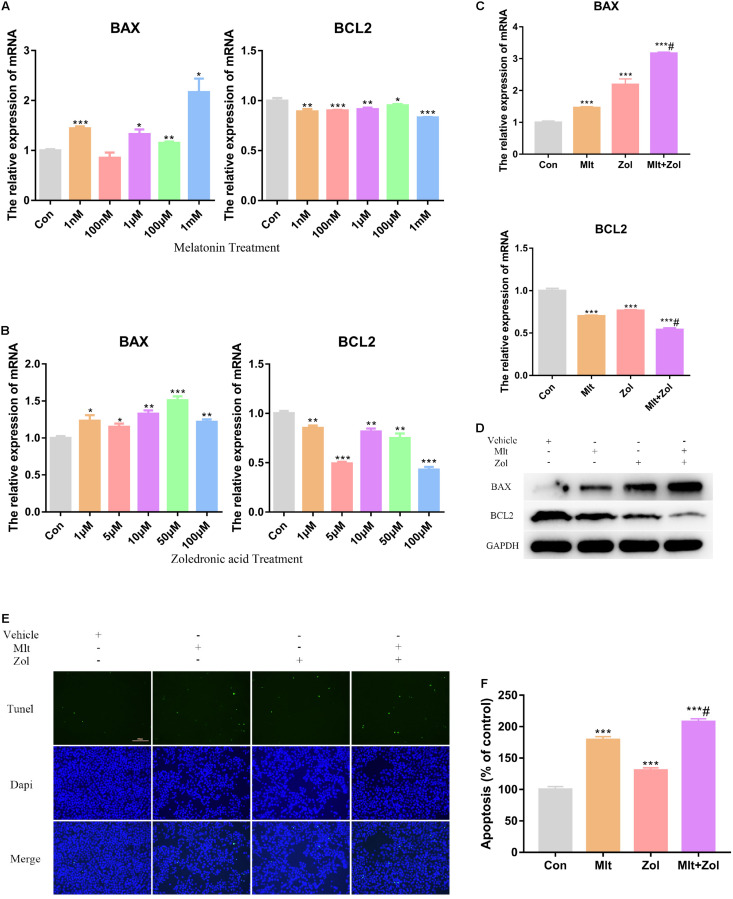
Mlt, Zol, and their combination promote the apoptosis of GCTB cells. **(A)** Relative mRNA levels of apoptosis markers’ genes (*BAX* and *BCL2*) in human GCTB cells treated with Mlt were measured by qRT-PCR. **(B)** Relative mRNA levels of apoptosis markers’ genes in human GCTB cells treated with Zol were measured by qRT-PCR. **(C,D)** Relative mRNA and protein levels of apoptosis markers in human GCTB cells treated with Mlt, Zol and Mlt + Zol were measured by qRT-PCR and western blot. **(E)** The TUNEL assay was used to detect the apoptosis of tumor cells after treatment with Mlt, Zol and their combination. **(F)** The quantitative results of apoptosis rate in each group in the TUNEL experiment. Con: control; Mlt: melatonin; Zol: zoledronic acid; *P < 0.05 vs. Con, **P < 0.01 vs. Con, ***P < 0.001 vs. Con, #*P* < 0.05 vs. Zol. Scale bars: 100 μm (magnification: 200×).

We explored the impact of different Mlt and Zol concentrations on tumor cell apoptosis. By exploring the mRNA expression of the apoptosis marker *BAX* and antiapoptotic marker *BCL2*, we found that exposure to Mlt and Zol resulted in the downregulation of the mRNA expression of *BCL2* and the upregulation of the mRNA expression of *BAX* (Figures 2A,B). Moreover, the mRNA and protein detection of these markers showed the combination of the two drugs showed a stronger apoptosis-promoting effect (Figures 2C,D). Furthermore, the TUNEL assay showed that Mlt, Zol, and their combination increased the apoptosis rate of tumor cells (Figures 2E,F). In summary, Mlt promoted the apoptosis of GCTB cells and can further enhance the positive effects of Zol.

### Mlt, Zol, and Their Combination Promote the Osteogenic Differentiation of GCTB Cells

**FIGURE 3:**
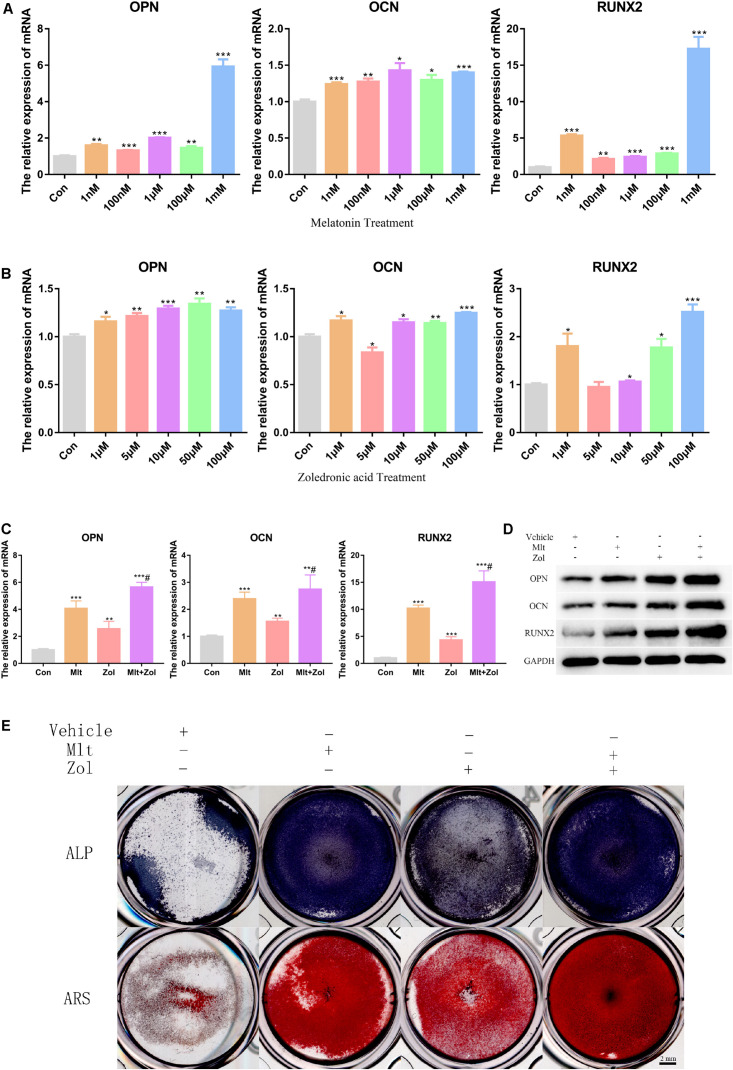
Mlt, Zol, and their combination promote osteogenic differentiation of GCTB cells. **(A)** Relative mRNA levels of osteogenic markers’ genes (*OPN*, *OCN*, and *RUNX2*) in human GCTB cells treated with Mlt were measured by qRT-PCR. **(B)** Relative mRNA levels of osteogenic markers’ genes in human GCTB cells treated with Zol were measured by qRT-PCR. **(C,D)** Relative mRNA and protein levels of osteogenic markers in GCTB cells treated with Mlt, Zol and Mlt + Zol were measured by qRT-PCR and western blot. **(E)** The osteogenic differentiation of GCTB cells in different groups were evaluated by ALP staining and Alizarin Red staining. Con: control; Mlt: melatonin; Zol: zoledronic acid; ALP: alkaline phosphatase; ARS: Alizarin Red staining; *P < 0.05 vs. Con, **P < 0.01 vs. Con, ***P < 0.001 vs. Con, #*P* < 0.05 vs. Zol. Scale bars: 2 mm.

In this part of the study, we explored the effects of different Mlt and Zol concentrations on the mRNA and protein expression of osteogenesis markers (OPN, OCN, and RUNX2) in tumor cells. We found that different concentrations of either Mlt or Zol promoted the mRNA expression of osteogenesis markers in GCTB cells, and the effect of a higher concentration was more obvious (Figures 3A,B). Moreover, the combination of the two drugs showed a stronger effect in promoting osteogenic differentiation (Figures 3C,D). ALP staining and ARS were then used to stain the GCTB cells after 7 days of drug treatment. We found that Mlt and Zol increased the ALP staining and ARS intensity of tumor cells; moreover, the combination of the two drugs provided a stronger differentiation-promoting effect (Figure 3E). In summary, the above results showed that Mlt promoted the osteogenic differentiation of tumor cells and the combined effect of Mlt and Zol was stronger than that of Zol alone.

### Mlt, Zol, and Their Combination Inhibit the Scratch Healing Ability of GCTB Cells

**FIGURE 4:**
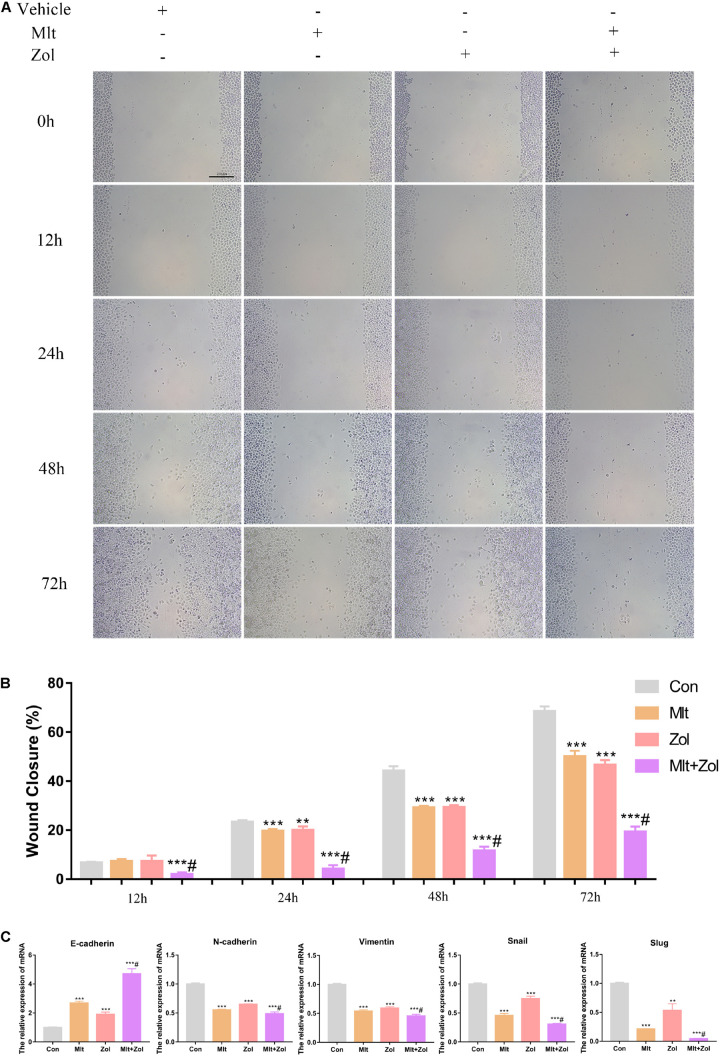
Mlt, Zol, and their combination inhibit the scratch healing ability of GCTB cells. **(A)** The scratch healing assay was used to detect the scratch healing ability of tumor cells after treatment with Mlt, Zol and the two drugs at 12 h, 24 h, 48 h and 72 h. **(B)** The quantitative results of the scratch healing experiment. **(C)** Relative mRNA levels of EMT markers (*E-cadherin*, *N-cadherin*, *Vimentin*, *Snail* and *Slug*) in GCTB cells treated with Mlt, Zol and Mlt + Zol were measured by qRT-PCR. Con: control; Mlt: melatonin; Zol: zoledronic acid; EMT: epithelial mesenchymal transition; **P < 0.01 vs. Con, ***P < 0.001 vs. Con, #*P* < 0.05 vs. Zol. Scale bars: 200 μm.

We explored the effects of Mlt, Zol, and their combination on the scratch healing ability of tumor cells. Using the tumor cell scratch test to detect the healing ability of tumor cells after drug treatments for 12, 24, 48, and 72 h, we found that Mlt, Zol, and their combination inhibited the scratch healing ability of tumor cells, and the combination of the two drugs showed a stronger inhibitory effect (Figure 4A). The quantitative results of scratch width at different time points are shown in Figure 4B. Moreover, Mlt and Zol treatment significantly upregulated expression of *E-cadherin* but downregulated *N-cadherin*, *Vimentin, Snail*, and *Slug* in GCTB cells (Figure 4C). In conclusion, Mlt inhibited the scratch healing ability of tumor cells, and the inhibitory effect of Mlt combined with Zol was stronger than that of Zol alone.

### Mlt, Zol, and Their Combination Inhibit the Migration and Invasion of GCTB Cells

**FIGURE 5:**
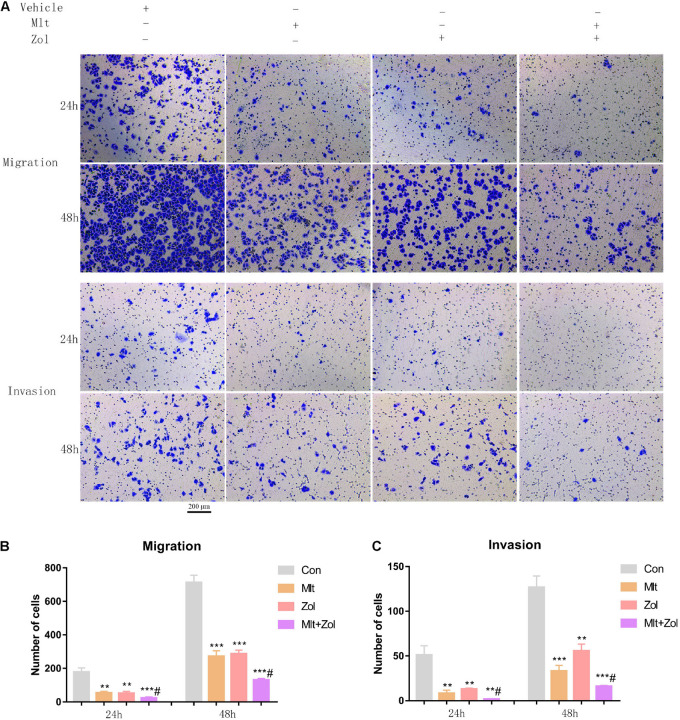
Mlt, Zol, and their combination inhibit the migration and invasion of GCTB cells. **(A)** The transwell assay was used to detect the migration and invasion ability of tumor cells after treatment with Mlt, Zol and the two drugs at 24 h and 48 h. **(B)** The quantitative results of transwell migration assay in panel **(A)**. **(C)** The quantitative results of transwell invasion assay in panel **(A)**. Con: control; Mlt: melatonin; Zol: zoledronic acid; **P < 0.01 vs. Con, ***P < 0.001 vs. Con, #*P* < 0.05 vs. Zol. Scale bars: 200 μm.

We explored the effect of Mlt, Zol, and their combination on the migration and invasion of tumor cells. Based on the transwell experiment performed after drug treatments for 24 and 48 h, we found that Mlt, Zol, and their combination inhibited the migration and invasion of GCTB cells. Furthermore, the combination of the two drugs showed a stronger inhibitory effect (Figure 5A). The quantitative results of the migration and invasion of GCTB cells are shown in Figures 5B,C. Above all, our results showed that Mlt inhibited the migration and invasion of tumor cells. Moreover, the inhibitory effect of Mlt combined with Zol was stronger than that of Zol alone.

### Mlt, Zol, and Their Combination Exert an Anti-GCTB Effect by Inhibiting Both the Hippo and Nuclear Factor Kappa B (NF-κB) Pathways

**FIGURE 6:**
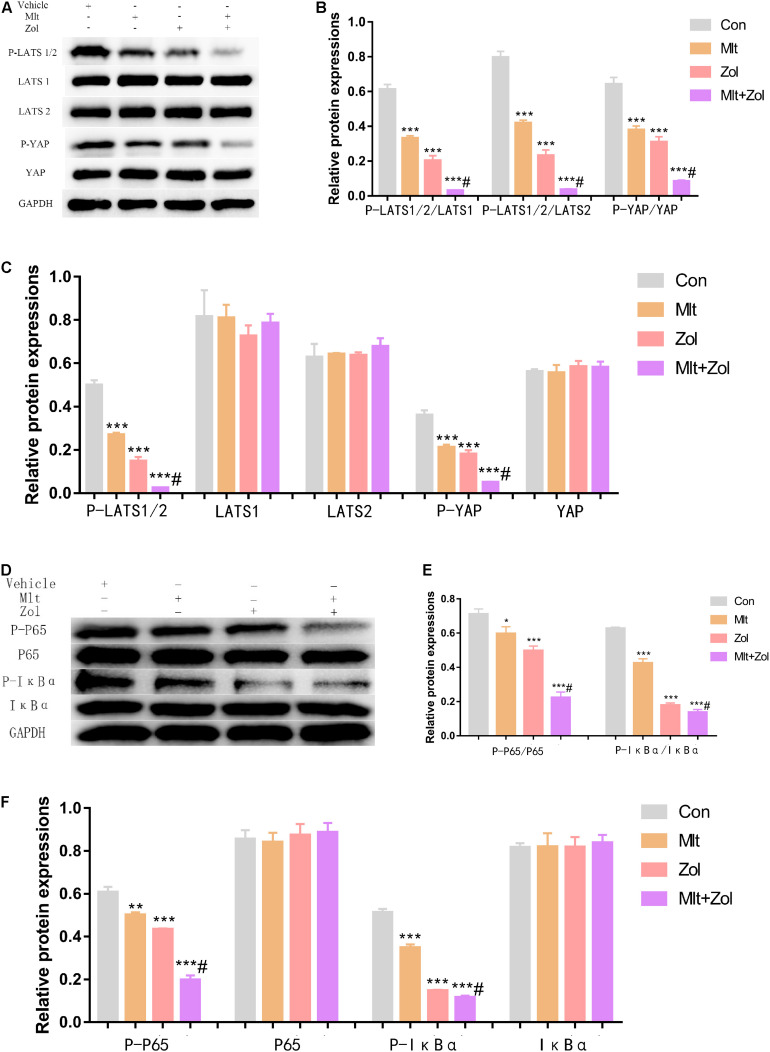
Mlt, Zol, and their combination inhibit the activation of both the Hippo and NF-κB pathway in GCTB cells. **(A)** P-LATS1/2, LATS1, LATS2, P-YAP, and YAP protein levels in GCTB cells treated with Mlt, Zol, Mlt + Zol. Western blot analysis was used to detect protein levels using GAPDH as the internal standard. **(B)** The ratio of relative protein expression of P-LATS1/2,P-LATS1/2 and P-YAP to relative protein expression of LATS1,LATS2 and YAP. **(C)** The ratio of relative protein expression of P-LATS1/2, LATS1, LATS2, P-YAP and YAP to relative protein expression of GAPDH. **(D)** P-P65, P65, P-IκBα, and IκBα protein levels in GCTB cells treated with Mlt, Zol and Mlt + Zol. Western blot analysis was used to detect protein levels using GAPDH as the internal standard. **(E)** The ratio of relative protein expression of P-P65 and P-IκBα to relative protein expression of P65 and IκBα. **(F)** The ratio of relative protein expression of P-P65, P65, P-IκBα, and IκBα to relative protein expression of GAPDH. Con: control; Mlt: melatonin; Zol: zoledronic acid; GAPDH: glyceraldehyde-3-phosphate dehydrogenase; *P < 0.05 vs. Con, **P < 0.01 vs. Con, ***P < 0.001 vs. Con, #*P* < 0.05 vs. Zol.

We explored the mechanisms corresponding to the antitumor effects of Mlt and Zol. Our results showed that Mlt, Zol, and their combination suppressed the phosphorylation level of the key molecules (LATS1, LATS2, and YAP) in the Hippo pathway and in the NF-κB pathway (P65 and IκBα) compared with the control group. In addition, the combination of the two drugs showed a stronger inhibition (Figures 6A,D). The protein quantitative results of each group were shown in Figures 6B,C,E,F. Moreover, we found that Mlt and Zol inhibited the entry of P65 into the nucleus and the expression of NF-κB pathway targeted genes (*MMP9*, *MMP13*, and *CCL2*). In addition, the combination of the two drugs showed a stronger inhibition (Supplementary Figures 2, 3). In summary, our results revealed that the therapeutic effect of Mlt and Zol can be achieved by inhibiting the activation of both the Hippo and NF-κB pathways.

### Mlt, Zol, and Their Combination Inhibit the Growth of Animal Tumors by Inhibiting Cell Proliferation and Promoting Cell Apoptosis

In this part of the research, we used GCTB cells for subcutaneous tumor formation in the inguinal area of nude mice and then treated the mice with Mlt, Zol, and their combination for 5 weeks to explore the effect of drug treatments on tumor growth.

**FIGURE 7:**
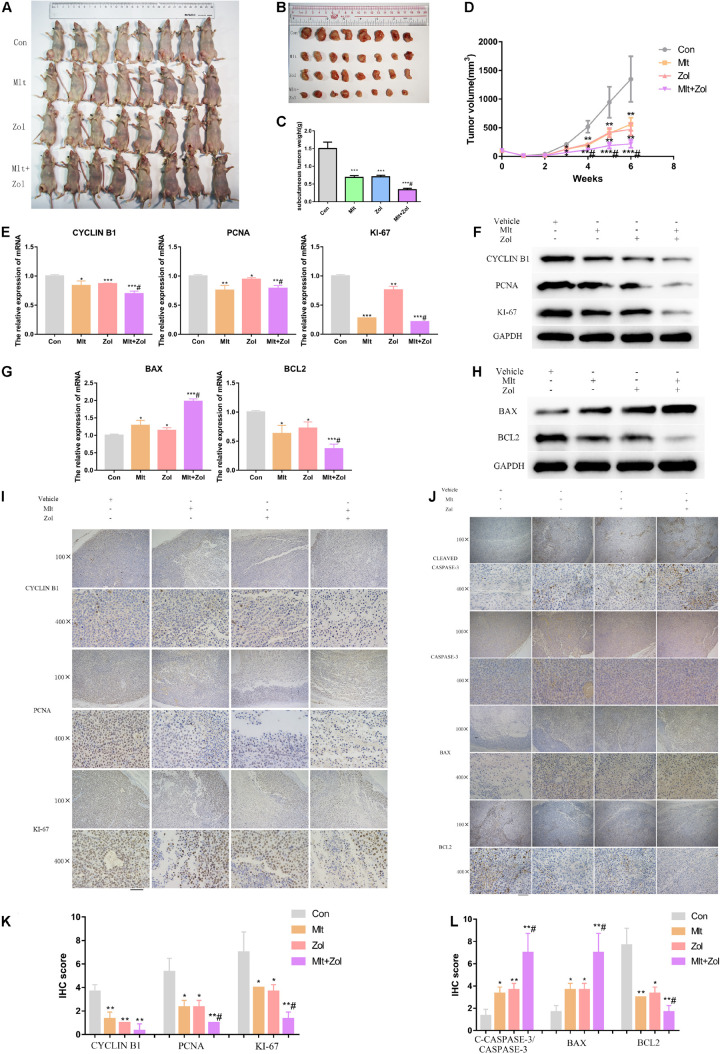
Mlt, Zol, and their combination inhibit the growth of animal tumors by inhibiting cell proliferation and promoting cell apoptosis. **(A,B)** Representative pictures of tumors in nude mice with different treatments after 5 weeks. **(C)** Tumor weight statistics of nude mice with different treatments after 5 weeks. **(D)** Tumor volume growth curves of nude mice in different groups after different treatments. **(E,F)** Relative mRNA and protein levels of proliferation markers (CYCLIN B1, PCNA, and KI-67) of tumors in each group were measured by qRT-PCR and western blot. **(G,H)** Relative mRNA and protein levels of apoptosis markers (BAX and BCL2) of tumors in each group were measured by qRT-PCR and western blot. **(I)** The proliferation markers’ proteins (CYCLIN B1, PCNA, and KI-67) of tumors in each group were measured by IHC staining. **(J)** The apoptosis proteins’ markers (CLEAVED CASPASE-3, CASPASE-3, BAX, and BCL2) of tumors in each group were measured by IHC staining. **(K)** The IHC staining intensity of proliferation markers (CYCLIN B1, PCNA, and KI-67) were scored. **(L)** The IHC staining intensity of apoptosis markers (CLEAVED CASPASE-3/CASPASE-3, BAX, and BCL2) were scored; Con: control; Mlt: melatonin; Zol: zoledronic acid; *P < 0.05 vs. Con, **P < 0.01 vs. Con, ***P < 0.001 vs. Con, #*P* < 0.05 vs. Zol. Scale bars: 200 μm (100× figures), 50 μm (400× figures).

First, the body weight statistics of mice and pathology images of liver, spleen, kidney and heart in nude mice after drug administration showed that Mlt and Zol treatment were safe to the mice (Supplementary Figure 4). After 5 weeks of drug administration, we found that Mlt, Zol, and their combination significantly inhibited the growth of tumor tissue in nude mice, with a stronger inhibitory effect than Zol alone (Figures 7A,B). The same finding was observed *via* tumor weight measurements in the animals (Figure 7C). In the process of drug treatment, we found that Mlt, Zol, and their combination inhibited the tumor size growth trend of nude mice in different groups. Again, the combination of the two drugs showed a stronger inhibitory effect (Figure 7D).

Subsequently, we performed the mRNA and protein detection of proliferation markers (CYCLIN B1, PCNA, and KI-67) and apoptosis markers (BAX and BCL2) in the tumor tissues of each mice group. Our results demonstrated that Mlt and Zol treatment inhibited the mRNA and protein levels of the cell proliferation markers and the antiapoptotic marker BCL2 while promoting the mRNA and protein level of the proapoptotic marker BAX. In addition, the combination of the two drugs showed a stronger treatment effect (Figures 7E–H). IHC staining was then performed on the tumor tissues of each group, and similar findings were obtained (Figures 7I,J). The quantitative results of IHC staining in each group were shown in Figures 7K,L. In conclusion, Mlt inhibited the growth of animal tumors by inhibiting cell proliferation and promoting cell apoptosis. Moreover, the inhibitory effect of Mlt combined with Zol was stronger than that of Zol alone.

## Discussion

The present study explored the effects of Mlt, a safe and powerful endocrine hormone (Lamberg, 1996; Macchi and Bruce, 2004; Buscemi et al., 2006; Hobson et al., 2018; Gurunathan et al., 2020) on the treatment of GCTB. We found that Mlt showed antitumor effects by inhibiting the proliferation, migration, and invasion of GCTB cells, in addition to promoting the apoptosis and osteogenic differentiation of tumor cells, which is similar to the effect of Zol. Moreover, Mlt combined with Zol showed stronger therapeutic effects than Zol alone. This therapeutic effect might be achieved by inhibiting the activation of both the Hippo and NF-κB pathways. To the best of our knowledge, no studies have explored the effect of Mlt on GCTB. This study is expected to provide a sufficient experimental and theoretical basis for using Mlt in the future treatment of GCTB.

In the present study, Mlt inhibited the proliferation of tumor cells and promoted cell apoptosis to inhibit the progress of GCTB. CCK-8 and TUNEL experiments showed that different concentrations of Mlt could inhibit the proliferation of tumor cells and promote cell apoptosis (Figures 1, 2). In addition, the results showed that exposure to Mlt resulted in the downregulation of proliferation markers (CYCLIN B1, PCNA, and KI-67) (Figures 1, 7) and the antiapoptotic protein BCL-2, as well as the upregulation of the proapoptotic protein BAX (Figures 2, 7). Moreover, the Cleaved CASPASE/CASPASE ratio was upregulated (Figure 7). Likewise, previous studies reported that Mlt can inhibit tumor cell proliferation and promote tumor cell apoptosis *via* different ways, thereby playing a therapeutic role in various tumors, including lung cancer, nerve tumors, gastrointestinal tumors, liver cancer, and others (Xu C. et al., 2013; Zhang et al., 2013; Borin et al., 2016; Nooshinfar et al., 2016; Bu et al., 2017; Chovancova et al., 2017; Yu et al., 2018; Liu et al., 2019; Ma et al., 2019; Mortezaee et al., 2019).

The migration and invasion capacities of tumor cells are important indicators to assess the malignancy and the risk of distant metastasis of tumor cells. Using the scratch wound and transwell assays, we found that Mlt inhibited the migration and invasion of GCTB cells (Figures 4, 5). The results of the current study were similar to previous studies. Mlt has been reported to be a possible inhibitor of tumor cell migration and invasion, such as in breast cancer, ovarian cancer, oral cancer, and osteosarcoma (Akbarzadeh et al., 2017; Gu et al., 2017; Liu et al., 2018; Qu et al., 2018; El-Sokkary et al., 2019). In summary, Mlt is expected to be utilized in the treatment of GCTB for inhibiting tumor cell proliferation, migration, and invasion, as well as for promoting apoptosis.

GCTB stromal cells are pathologically regarded as primary neoplastic cells and are known as incompletely differentiated preosteoblasts. Therefore, promoting stromal cells in GCTB to differentiate into mature osteoblast cells may stop tumor growth and recurrence (Steensma et al., 2013; Yang et al., 2013; Lau et al., 2020). Lau et al. (2020) found that simvastatin could promote the osteogenic differentiation of GCTB stromal cells to stop tumor growth and recurrence. Lutsik et al. (2020) found that a mutation in *H3F3A* occurred in differentiating mesenchymal stem cells (MSCs) and was associated with an impaired osteogenic differentiation, which is contributed to the development of GCTB. In addition, studies have shown that Mlt promoted the osteogenic differentiation of MSCs and osteogenic precursor cells MC3T3-E1 (Son et al., 2014; Jiang et al., 2019). However, prior research has not ascertained whether Mlt can promote the osteogenic differentiation of GCTB cells. In the present study, we explored the effect of Mlt on the osteogenic differentiation of tumor cells by detecting osteogenic markers (OPN, OCN, and RUNX2) and staining related to osteogenic differentiation (ALP staining and ARS). We found that Mlt could effectively promote the osteogenic differentiation of tumor cells, thereby delaying the malignant progression of GCTB (Figure 3).

Zol is a commonly used adjuvant for GCTB treatment (Chen et al., 2015; Nishisho et al., 2015; Li et al., 2019; Luna et al., 2020). Studies have shown that Zol can inhibit the proliferation and migration of GCTB while promoting the osteogenic differentiation and apoptosis of tumor cells (Cheng et al., 2004; Balke et al., 2010; Yang et al., 2013; Kundu et al., 2018; Li et al., 2018; Dubey et al., 2019; Shibuya et al., 2019). Our study showed similar findings, corroborating the inhibitory effect of Zol on GCTB. Moreover, we explored the therapeutic effects of Zol in combination with Mlt on GCTB cells. Both *in vitro* and *in vivo* experiments have fully demonstrated that the combination of these two drugs had a stronger therapeutic effect than Zol alone (Figures 1–5, 7), which offers a new direction for the future treatment of GCTB.

To further explore the mechanisms of the antitumor effects of Mlt and Zol, we detected changes in the signaling pathway related to the therapeutic effects of Mlt and Zol. The NF-κB family plays an indispensable role in the survival of many tumors, usually enabling them to metastasize and develop resistance to treatment (Hu et al., 2016). Our results showed that Mlt combined with Zol inhibited the activation of NF-κB pathway by reducing the phosphorylation levels of P65 and IκBα proteins, and then inhibited the entry of P65 into the nucleus and the expression of NF-κB pathway targeted genes (*MMP9*, *MMP13*, and *CCL2*) (Figure 6 and Supplementary Figures 2, 3). These findings confirm that the inhibition of the NF-κB pathway is necessary for the successful treatment of GCTB mediated by the combination of Mlt and Zol.

The Hippo pathway also plays an important role in tumor development. The mutation and expression changes of its core components (MST1/2, LATS1/2, YAP, and TAZ) can promote the migration, invasion, and malignant progression of cancer cells (Maugeri-Sacca and De Maria, 2018; Han, 2019). Some researchers have proposed that the regulation of the Hippo pathway could be applied to the treatment of tumors (Cao and Huang, 2017). However, research has not confirmed whether the Hippo pathway also plays an important role in the development of GCTB. In the present study, we found that the activation of the Hippo pathway was significantly inhibited after treatment with Mlt and Zol, suggesting that Mlt and Zol may confer an antitumor effect by inhibiting the activation of this pathway (Figure 6). Therefore, these results suggest that Hippo pathway inhibitors may also be used to treat GCTB, a possibility that requires further research for verification.

There are several studies on crosstalk between the Hippo and NF-kB pathways. Wang et al. (2018) demonstrated that the proteasome activator REGγ could promote the activation of the Hippo and NF-kB pathways to promote colon cancer. Tang et al. (2021) found that titanium ions induced the activation of the Hippo pathway and then activated the NF-κB pathway, which finally upregulated the migration of macrophages. In addition, Yap, the core downstream effector of the Hippo signaling cascade, may regulate the activation of the NF-kB pathway in different cells, such as the MC3T3-E1 cells, undifferentiated pleomorphic sarcoma cells, HepG2 cells, and chondrocytes (Perumal et al., 2017; Deng et al., 2018; Rivera-Reyes et al., 2018; Yang et al., 2020). In our study, we found that Mlt combined with Zol inhibited the activation of both the Hippo and NF-κB pathways by reducing the phosphorylation levels of LATS1/2, YAP, P65 and IκBα proteins, which exerted an anti-GCTB effect (Figure 6). These findings confirmed that the inhibition of both the Hippo and NF-κB pathways was necessary for the successful treatment of GCTB.

Our study had several limitations. First, our study on the underlying mechanisms was not sufficiently complete. We found that after the Mlt and Zol treatment, the activation of the Hippo and NF-κB pathways was suppressed, with the reduced phosphorylation of LATS1, LATS2, YAP, P65, and IκBα. Besides, they inhibited the entry of P65 into the nucleus and the expression of NF-κB pathway targeted genes (*MMP9*, *MMP13*, and *CCL2*). However, whether the two pathways participate in crosstalk remains to be clarified. Second, we only use one GCTB cell line in this research, and more GCTB cell lines need to be proved in our follow-up in-depth studies. Third, this was a preliminary exploration study at the cellular and animal levels. Thus, clinical trials are still needed to better verify the therapeutic effects of Mlt on GCTB.

In summary, the current study demonstrated that Mlt could effectively inhibit the proliferation, migration, and invasion of GCTB cells, as well as promote the apoptosis and osteogenic differentiation of tumor cells. In addition, a stronger antitumor effect was observed when Mlt was combined with Zol treatment. This therapeutic effect might be achieved by inhibiting the activation of both the Hippo and NF-κB pathways. Therefore, this study suggests that Mlt can be a new treatment for GCTB, which could further enhance the antitumor effect of Zol.

## Data Availability Statement

The raw data supporting the conclusions of this article will be made available by the authors, without undue reservation.

## Ethics Statement

The animal study was reviewed and approved by The Forevergen Biosciences Experimental Animal Ethics Committee.

## Author Contributions

ZZ and DH designed the experiments. XW, PS, YK, CX, JQ, JW, and PS conducted the experiments. XW and PS acquired the data. XW, PS, ZZ, and DH analyzed the data. XW, ZZ, and DH wrote the manuscript. All authors read and approved the final manuscript.

## Conflict of Interest

The authors declare that the research was conducted in the absence of any commercial or financial relationships that could be construed as a potential conflict of interest.

## Publisher’s Note

All claims expressed in this article are solely those of the authors and do not necessarily represent those of their affiliated organizations, or those of the publisher, the editors and the reviewers. Any product that may be evaluated in this article, or claim that may be made by its manufacturer, is not guaranteed or endorsed by the publisher.
