# Supplementary material for: Combination of Melatonin and Zoledronic Acid Suppressed the Giant Cell Tumor of Bone in vitro and in vivo
Source: Front Cell Dev Biol. 2021 Aug 10;9:690502. doi: 10.3389/fcell.2021.690502 (PMC8382950; doi:10.3389/fcell.2021.690502)
Supplement: Supplementary file 1 [file Data_Sheet_1.docx]

**Combination of melatonin and zoledronic acid suppressed the giant cell tumor of bone in vitro and in vivo**

**Supplementary Figures and figure legends**

**Supplementary Fig. 1**

**
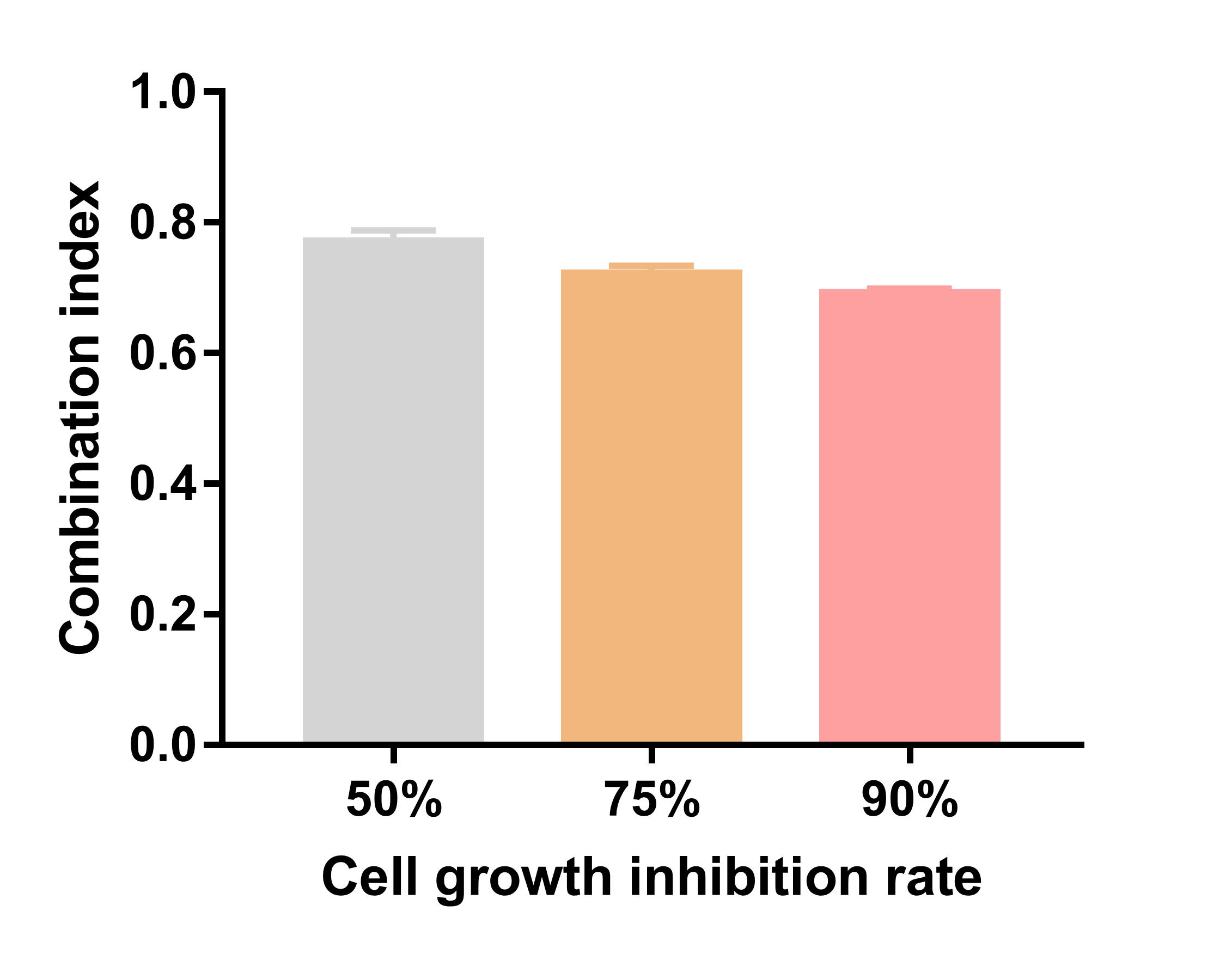
**

**Supplementary Fig.** **1** The combination index（CI）of Mlt and Zol in different cell growth inhibition rate (50%, 75% and 90%). Note: 0.9≤CI≤1.1 indicates additive effect, 0.8≤CI<0.9 indicates low synergistic effect, 0.6≤CI<0.8 indicates moderate synergistic effect, 0.4≤CI<0.6 indicates high synergistic effect, and 0.2≤CI<0.4 indicates strong synergistic effect.

**Supplementary Fig.** 2


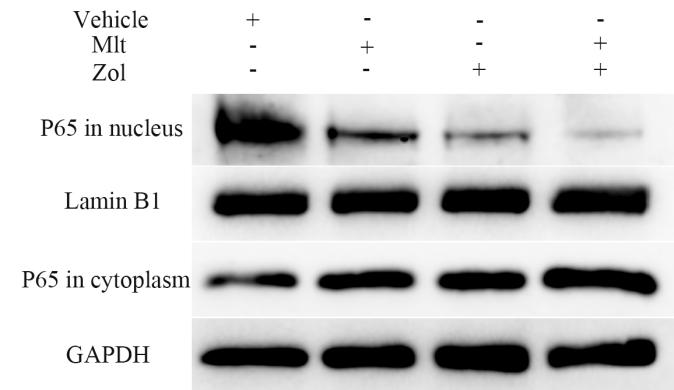


**Supplementary Fig.** **2** The expression of NF-κB p65 in nucleus and cytoplasm of GCTBs through western blot.

**Supplementary Fig. 3**


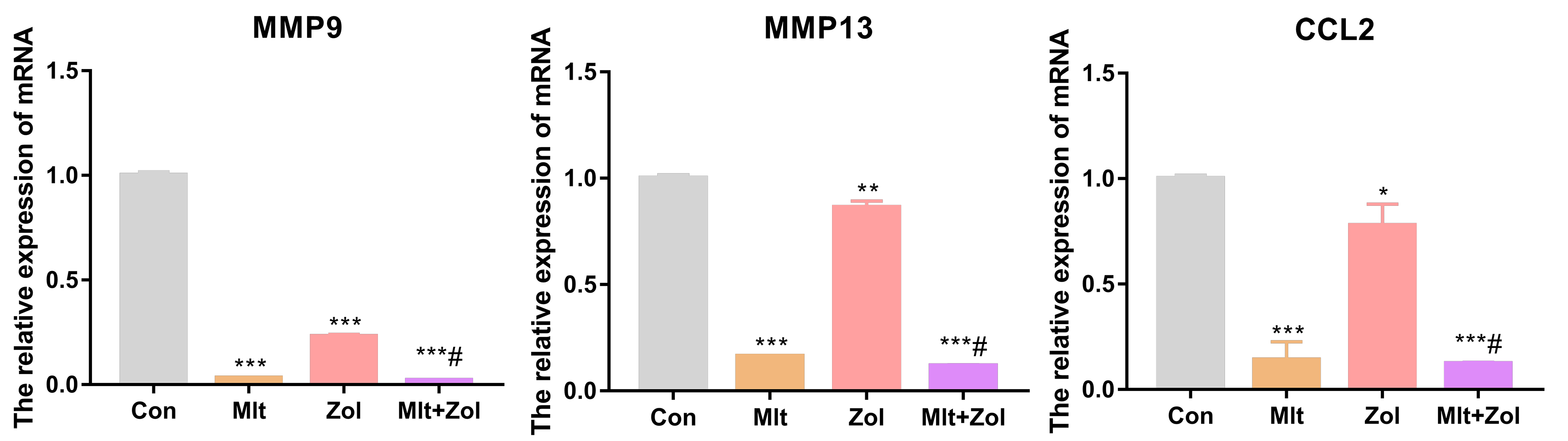


**Supplementary Fig. 3** The mRNA expression of NF-κB pathway-targeted genes.

Relative mRNA levels of NF-κB pathway-targeted genes (*MMP9*, *MMP13*, and *CCL2*) in GCTB cells treated with Mlt, Zol and Mlt+Zol were measured by qRT-PCR.

**Supplementary Fig. 4**

**
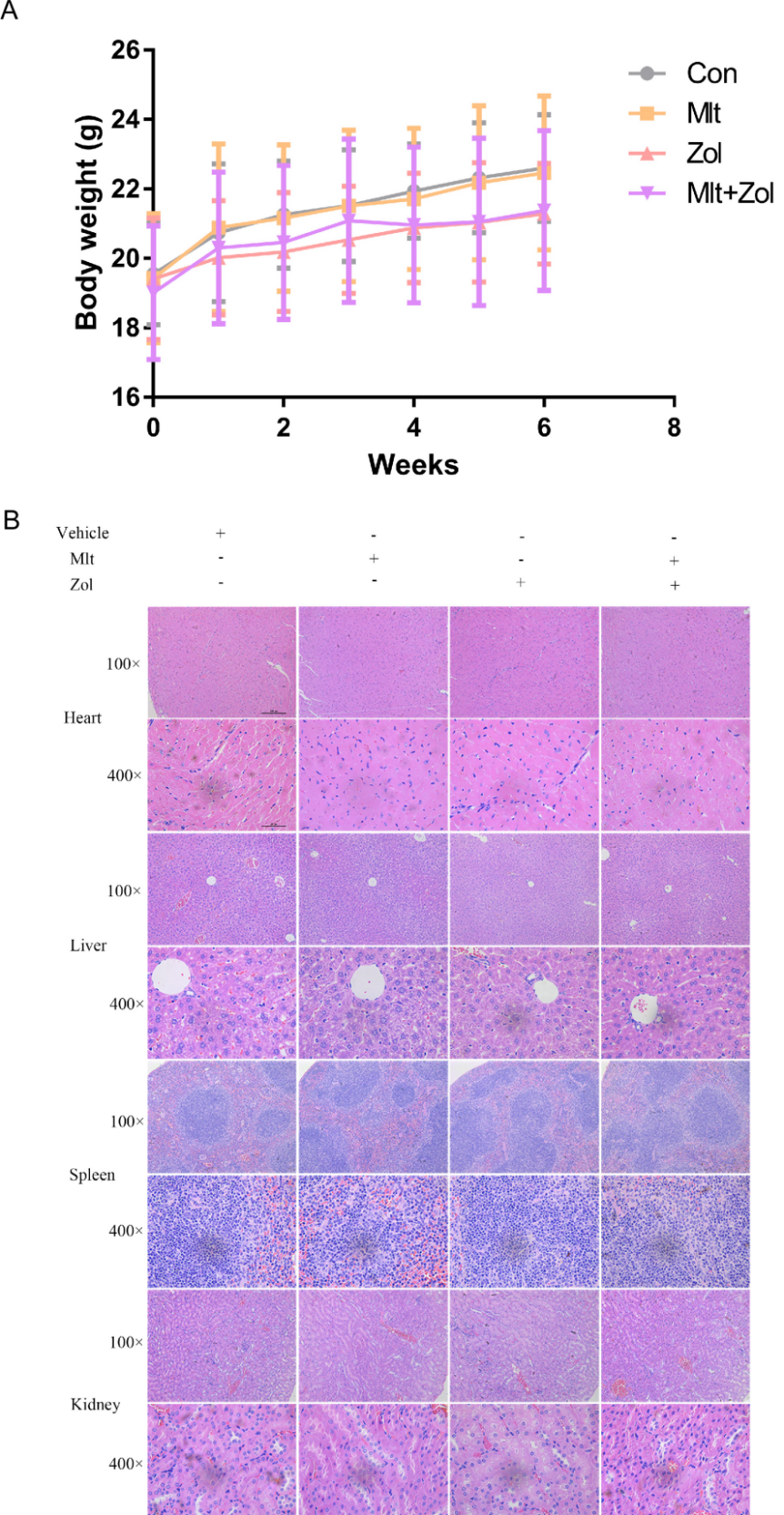
**

**Supplementary Fig.** 4 The body weight statistics of mice and pathology images of liver, spleen, kidney and heart in nude mice. (**A**) Body weight statistics of nude mice with different treatments after 5 weeks. (**B**) Pathology images of liver, spleen, kidney and heart in nude mice with different treatments after 5 weeks. Scale bars: 200 μm (100× figures), 50 μm (400× figures).
